# Effect of lycopene on cell viability and cell cycle progression in human cancer cell lines

**DOI:** 10.1186/1475-2867-12-36

**Published:** 2012-08-06

**Authors:** Anderson Junger Teodoro, Felipe Leite Oliveira, Nathalia Balthazar Martins, Guilherme de Azevedo Maia, Renata Brum Martucci, Radovan Borojevic

**Affiliations:** 1 Laboratory of Nutritional Biochemistry, Program of Food and Nutrition, UNIRIO, Rio de Janeiro, Brazil; 2 Laboratory of Cell Proliferation and Differentiation, Institute of Biomedical Sciences, Federal University of Rio de Janeiro, Rio de Janeiro, Brazil; 3 Excellion Biomedical Services, Petrópolis, Rio de Janeiro, Brazil; 4 Departamento de Tecnologia de Alimentos, Universidade Federal do Estado do Rio de Janeiro, Escola de Nutrição, CEP 22290-240, Rio de Janeiro, RJ, Brazil

**Keywords:** Lycopene, Cancer, Bioactive compounds, Cell cycle

## Abstract

**Background:**

Lycopene, a major carotenoid component of tomato, has a potential anticancer activity in many types of cancer. Epidemiological and clinical trials rarely provide evidence for mechanisms of the compound’s action, and studies on its effect on cancer of different cell origins are now being done. The aim of the present study was to determine the effect of lycopene on cell cycle and cell viability in eight human cancer cell lines.

**Methods:**

Human cell lines were treated with lycopene (1–5 μM) for 48 and 96 h. Cell viability was monitored using the method of MTT. The cell cycle was analyzed by flow cytometry, and apoptotic cells were identified by terminal deoxynucleotidyl transferase-mediated dUTP nick labeling (TUNEL) and by DAPI.

**Results:**

Our data showed a significant decrease in the number of viable cells in three cancer cells lines (HT-29, T84 and MCF-7) after 48 h treatment with lycopene, and changes in the fraction of cells retained in different cell cycle phases. Lycopene promoted also cell cycle arrest followed by decreased cell viability in majority of cell lines after 96 h, as compared to controls. Furthermore, an increase in apoptosis was observed in four cell lines (T-84, HT-29, MCF-7 and DU145) when cells were treated with lycopene.

**Conclusions:**

Our findings show the capacity of lycopene to inhibit cell proliferation, arrest cell cycle in different phases and increase apoptosis, mainly in breast, colon and prostate lines after 96 h. These observations suggest that lycopene may alter cell cycle regulatory proteins depending on the type of cancer and the dose of lycopene administration. Taken together, these data indicated that the antiproliferative effect of lycopene was cellular type, time and dose-dependent.

## Background

Diets high in fruits and vegetables are associated with reduced rates of cancer and coronary heart disease. Lycopene, a major carotenoid component of tomato, exhibited potential anticancer activity in many types of cancer [1,2]. Epidemiological studies reported statistically significant inverse association between tomato consumption and risk of several types of cancer such as lung, prostate and colon cancer [3-5].

Despite epidemiological and small clinical trial evidence suggesting a possible protective effect of lycopene, the mechanism of its action including cell cycle arrest and induction of apoptosis, remain poorly understood [6,7]. Lycopene has been proposed to negatively affect cancer cells or development of cancer by modulating cell cycle progression and cell proliferation. It has an inhibitory effect on DNA synthesis, initiating up-regulation of gap-junction proteins and a reduction of local androgen signaling, impact IGIF-1 signaling, antioxidant activity and induction of apoptotic cell death, indicating that carotenoids are promising chemopreventive agents, with several cellular effects which are both genomic and non-genomic. [8,9]. Although there is significant evidence supporting the action of lycopene as a potent antioxidant, a number of other potential mechanisms through which tomato products providing lycopene may reduce the risk for cancer can be proposed [10-12].

Lycopene has antiproliferative effect on prostate and breast cancer cell lines. In breast cancer models, reduced expression of cell cycle regulatory proteins, such as cyclins D1 and E and the cyclin-dependent kinases 2 and 4, as well as suppression of insulin-like growth factor (IGF-I) action have been correlated with lycopene’s effects on proliferation [13,14]. Other studies reported that lycopene had limited effect on cell proliferation of cancerous and noncancerous cell lines in an in vitro system with doses within the physiological range, but only rare studies reported no effect of lycopene on cell proliferation [15].

Deregulated cell cycle is one of the major hallmarks of cancer cells. These cells may lose the ability to regulate the cell cycle and control their rate of proliferation. A rate-limiting step in the cell cycle that is often disturbed in cancer is the progression of cells through the first gap (G1) phase [16]. Recently, several reports show that lycopene can induce cell cycle arrest at the G1 phase. Park et al. [17] reported that the growth of human hepatoma cells (Hep3B) was inhibited 20–50% by lycopene at physiological concentrations as low as 0.2 μM. Lycopene was found to induce G0/G1 arrest and S phase block. In a similar study with the human prostate cancer cell lines LNCaP and PC3, Ivanov et al. [18] found that lycopene induced mitotic arrest at the G0/G1 phase.

Although, the role of lycopene in prevention of prostate cancer has been studied more extensively, human studies with tomatoes and lycopene on cancer affecting other organs are now being undertaken. The majority of studies on cell cycle were carried out in prostate and breast cancer cell lines after 48 h of lycopene treatment. The aim of the present study was to determine the effect of lycopene on cell cycle and cell proliferation in eight different human cancer cell lines at different time points.

## Results

### Effect of lycopene on number of viable cells in culture

All cells lines were shown to have normal growth characteristics expected under standard in vitro culture conditions. Previous studies reported that lycopene formulated as 10% WS granules was not toxic. Hereafter, all the results refer to lycopene with this vehicle, already used in our previous studies in vitro [19].

The plating of cancer cells lines was followed by 24 h recovery, and cell were subsequently incubated with 1, 3 or 5 μM lycopene during 24, 48 and 96 h. Lycopene was internalized into all cell lines, and reached the highest intracellular concentrations in HT-29 and T-84 lines. We tested the effect of lycopene on viability of the same cell lines in higher concentrations, but the results showed the same characteristics of inhibition from 5 to 40 micromolar. The mean intracellular lycopene concentration was 0.2-1.5 fmol/L, no increase was observed from 24 to 48 h, and only a slight increase was noted after 96 hours (data not shown).

**Figure 1:**
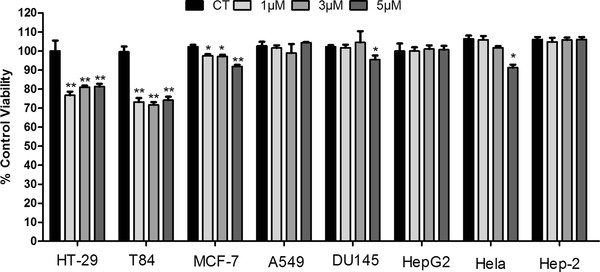
**Effect of lycopene on cell lines viability after 48 h of exposure using MTT assays.** The representative experiment is a mean ± error standard and a significant difference between each lycopene concentration and the control evaluated using the Tukey–Kramer Multiple Comparison test, *p < 0.05; **p < 0.01.

We used the MTT assay to monitor the cell viability. After treatment for 48 h, a significant decrease in the number of viable cells was observed in three cancer cells lines as compared to controls, reaching 30% reduction in HT-29 and T84 cells and 10% in MCF-7 cells. A discreet effect was observed in DU 145 and HeLa cells only with 5 μM lycopene. No effect was seen in the other cells (Figure 1).

**Figure 2:**
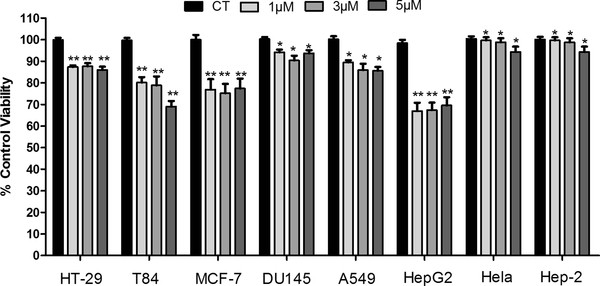
**Effect of lycopene on cell lines viability after 96 h of exposure using MTT assays.** The representative experiment is a mean ± error standard and a significant difference between each lycopene concentration and the control evaluated using the Tukey–Kramer Multiple Comparison test; *p < 0.05; **p < 0.01.

After 96 h, lycopene caused a significant modification of cell viability in six out of eight cell lines. Lycopene treatment inhibited in 25% and 30% cell viability of MCF-7 and HepG2 cells, respectively. The lowest cell viability reduction was observed in Hela and Hep-2 cells (Figure 2). Taken together, these data indicate that the effect of lycopene was cell-specific and time dependent, and that this effect required a relatively long incubation time in the majority of cell lines.

### Effect of lycopene on cell cycle progression

In order to monitor the influence of lycopene on cell cycle, we treated cells with lycopene for 48 and 96 h and quantified the percentage of cells in different cell cycle phases (Tables 1 and 2).

**Table 1:** Effect of lycopene (1-5 μM) on cell cycle progression in different human cancer cell lines after 48 hours

**Cell Lines**	**Cell Cyle phases**	**CT**	**1 μM**	**3 μM**	**5 μM**
**HT-29**	**G0/G1**	47.40 ± 0.57	61.65 ± 3.61*	55.10 ± 1.41*	59.05 ± 7.42*
	**S**	21.30 ± 4.53	19.70 ± 0.42	19.75 ± 0.49	20.70 ± 0.85
	**G2/M**	18.55 ± 3.61	13.70 ± 1.84	18.90 ± 0.42	12.45 ± 3.18
**T84**	**G0/G1**	42.20 ± 0.39	40.31 ± 0.40	41.10 ± 0.71	44.96 ± 2.76*
	**S**	10.93 ± 0.13	11.90 ± 0.60	10.25 ± 1.07	10.60 ± 0.45
	**G2/M**	33.00 ± 0.09	35.03 ± 1.74	33.90 ± 0.88	31.53 ± 0.81*
**MCF-7**	**G0/G1**	29.45 ± 2.05	29.15 ± 1.48	32.65 ± 0.49*	33.20 ± 1.13*
	**S**	17.00 ± 4.24	18.05 ± 2.90	16.75 ± 3.18	17.35 ± 0.92
	**G2/M**	36.50 ± 0.71	34.50 ± 0.71	16.90 ± 1.27*	22.40 ± 1.98*
**A549**	**G0/G1**	55.64 ± 0.28	57.84 ± 0.77	56.34 ± 0.14	58.82 ± 0.33*
	**S**	14.85 ± 0.05	13.99 ± 0.16	14.32 ± 0.23	13.43 ± 0.45
	**G2/M**	31.01 ± 0.28	29.58 ± 0.73	30.82 ± 0.02	28.86 ± 0.81
**DU145**	**G0/G1**	47.73 ± 2.21	48.49 ± 0.81	46.46 ± 4.21	56.14 ± 1.33*
	**S**	13.52 ± 1.12	12.46 ± 0.44	11.47 ± 1.27	9.10 ± 0.76
	**G2/M**	36.76 ± 0.76	36.24 ± 0.35	36.14 ± 1.61	31.20 ± 0.54*
**HepG2**	**G0/G1**	28.70 ± 2.40	29.75 ± 0.35	25.20 ± 2.55	28.45 ± 0.78
	**S**	17.80 ± 2.55	16.85 ± 0.21	16.20 ± 1.70	16.20 ± 1.13
	**G2/M**	27.95 ± 1.34	26.82 ± 0.99	25.30 ± 4.53	25.70 ± 1.84
**Hela**	**G0/G1**	62.42 ± 0.31	62.48 ± 0.08	63.49 ± 1.33	63.59 ± 2.26
	**S**	10.35 ± 0.28	9.02 ± 0.59	8.19 ± 0.24	7.86 ± 0.33
	**G2/M**	25.87 ± 0.27	26.36 ± 0.28	24.90 ± 0.32	26.19 ± 0.12
**Hep2**	**G0/G1**	64.80 ± 0.25	67.02 ± 0.54	66.98 ± 0.00	63.97 ± 0.50
	**S**	6.83 ± 0.05	6.75 ± 0.38	7.31 ± 0.08	7.48 ± 0.72
	**G2/M**	28.56 ± 0.20	26.45 ± 0.66	26.98 ± 1.28	28.99 ± 0.16

Results are expressed as the percentage of total cells. Data represent mean ± SD values of triplicate experiments. Tukey–Kramer Multiple Comparison test; *p < 0.05; **p < 0.01

**Table 2:** Effect of lycopene (1-5 μM) on cell cycle progression in different human cancer cell lines after 96 hours

**Cell Lines**	**Cell Cycle phase**	**CT**	**1 μM**	**3 μM**	**5 μM**
**HT-29**	**G0/G1**	77.14 ± 0.02	84.37 ±0.10*	40.29 ± 5.40**	46.07 ± 0.68**
	**S**	8.31 ± 0.45	4.40 ± 1.06	19.59 ± 4.02*	9.30 ± 0.47
	**G2/M**	13.41 ± 0.70	10.17 ± 0.32*	34.72 ± 0.86**	40.03 ± 0.38**
**T84**	**G0/G1**	28.14 ± 0.01	41.84 ± 1.53**	32.97 ± 0.28**	36.06 ± 0.06**
	**S**	17.86 ± 1.03	17.52 ± 0.05	18.85 ± 0.20	19.39 ± 3.93
	**G2/M**	43.67 ± 0.01	38.51 ± 1.48*	45.64 ± 0.51	50.23 ± 0.01*
**MCF-7**	**G0/G1**	17.10 ± 1.56	22.45 ± 0.78*	19.30 ± 0.99*	20.20 ± 1.13*
	**S**	26.25 ± 0.35	26.95 ± 0.07	24.15 ± 1.20	22.95 ± 1.48
	**G2/M**	40.50 ± 0.71	33.85 ± 1.20*	30.20 ± 0.28*	24.95 ± 0.07**
**A549**	**G0/G1**	65.82 ± 2.62	63.48 ± 4.47	66.62 ± 3.59	61.54 ± 1.14
	**S**	14.10 ± 3.29	13.61 ± 2.64	11.55 ± 2.67	14.90 ± 0.36
	**G2/M**	21.31 ± 0.83	24.56 ± 2.21*	23.40 ± 1.41*	25.18 ± 0.73*
**DU145**	**G0/G1**	56.24 ± 7.90	46.12 ±15.97	25.99 ± 7.43**	16.76 ± 1.61**
	**S**	12.19 ± 3.79	16.36 ± 3.25	8.02 ± 2.14	8.57 ± 1.70
	**G2/M**	29.89 ± 3.99	31.26 ± 5.85	56.97 ± 6.24**	55.93 ± 5.53**
**HepG2**	**G0/G1**	59.68 ± 0.09	47.17 ± 4.18*	23.23 ± 0.59**	19.91 ± 5.82**
	**S**	12.33 ± 0.86	10.68 ± 2.74	7.04 ± 0.52*	7.91 ± 2.71
	**G2/M**	26.39 ± 0.41	36.63 ± 7.25*	57.84 ± 1.53**	59.49 ±7.52**
**Hela**	**G0/G1**	62.42 ± 0.01	62.48 ± 0.08	63.49 ± 1.33	63.59 ± 2.26
	**S**	10.35 ± 0.18	9.02 ± 0.59	8.19 ± 0.24	7.86 ± 0.33
	**G2/M**	25.87 ± 0.27	26.36 ± 0.28	24.90 ± 0.32	26.19 ± 0.12
**Hep-2**	**G0/G1**	62.78 ± 4.35	69.50 ± 4.65	63.39 ± 0.07	61.83 ± 7.97
	**S**	18.73 ± 2.57	12.72 ± 3.64	16.79 ± 0.73	17.77 ± 5.70
	**G2/M**	20.66 ± 1.48	18.65 ± 1.92	20.31 ± 0.72	20.53 ± 2.57

Results are expressed as the percentage of total cells. Data represent mean ± SD values of triplicate experiments. Tukey–Kramer Multiple Comparison test; *p < 0.05; **p < 0.01

After 48 h, the three cell lines that have undergone major changes in cell viability were also those that had major changes in the percentages of cells in different cell cycle phases. In HT-29 cells, lycopene induced an increase in the percentage of cells in G0/G1. At 5 μM, a small inhibition of cell cycle progression was also observed in T84 cells, followed by decreased cell numbers in G2/M phase (p < 0.05). Moreover, treatment with lycopene (3 and 5 μM) for 48 h in MCF-7 cells resulted in G0/G1 arrest, showed by the accumulation of cells in G0/G1 with concomitant decrease in in the G2/M phase (p < 0.05) (Table 1).

A549 and DU145 lines showed minor changes in the distribution profile in cell cycle phases, with an increase of cells in G0/G1 phase and a decrease in G2/M phase (Table 1), only with the highest dose of lycopene (5 μM) (p < 0.05). No change was observed after exposure to lycopene in Hep-2, HeLa and HepG2 lines (Table 1), confirming the results of cell viability (Figure 1).

After 96 h, significant changes were observed in cell cycle of cancer cells treated with lycopene as compared to controls (Table 2). HT-29 cells presented an increase in G2/M phase when treated with 3 and 5 μM lycopene, with a decrease in the G0/G1 phase (p < 0.05). Lycopene also caused T84 cells to accumulate in the G0/G1 phase, regardless of the dose. Treatment with 1 and 5 μM lycopene led to an increase in the percentage of cells in G2/M phase.

In MCF-7 cells, an increase of cells in G0/G1 phase and a decrease in G2/M phase were observed after 48 h treatment with lycopene (p < 0.05). A549 cells showed a small increase of cells in G2/M phase (p < 0.05), with no significant difference among the different concentrations of lycopene.

The DU145 cell line showed an increase in cells retained in the G2/M phase, followed by a decrease of cells in G0/G1 phase when treated with 3 and 5 μM of lycopene (p < 0.01). This same was observed in Hep-G2 line at all concentrations, being more pronounced with higher concentrations (3–5 μM) of lycopene, coupled with a decrease in the percentage of cells in the phase S (p < 0.05). Similar to the data on cell viability, lycopene did not change the growth profile of two cell lines (Hela and Hep-2) (p > 0.05).

### Apoptosis

**Figure 3:**
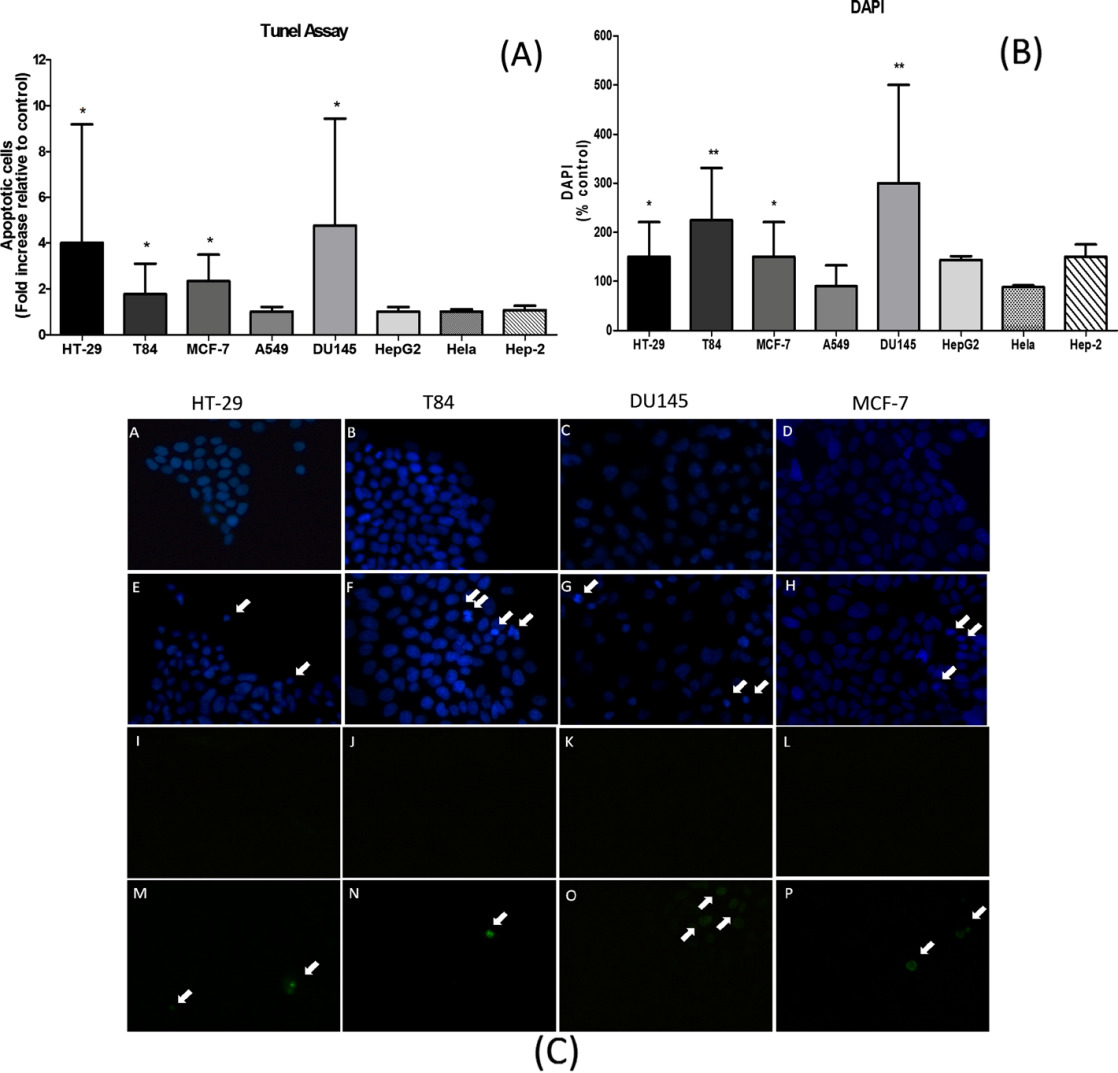
**Apoptosis-inducing activity of lycopene in human cancer cells.** (**A**) Human cancer cells incubated with lycopene (3 μM) for 96 h were analyzed by the TUNEL assay. TUNEL-positive cells were measured by fluorescence microscope. (**B**) Lycopene-treated human cancer cells were then stained with DAPI (4′,6-diamidino-2-phenylindole) for 3 min. Chromatin condensation was observed using fluorescence microscopy. Arrows in the micrographs indicate areas of chromatin condensation. Percentage of cells undergoing apoptosis was determined by counting apoptotic nuclei of DAPI-stained cells. ND, not detected. Values are means ± SD (n = 3). Bars with asterisks are significantly different from controls (p > 0.05). (**C**) Detection of apoptotic cells (in green) by TUNEL method in human cancer cells. The cells were treated (M-P) or not (**I-L**) with lycopene for 48 h. Nuclei were counterstained with DAPI (blue) (**A-H**). Chromatin condensation was observed in treated cells (**E-H**).

Quantification of apoptosis can be a useful measure of cancer cell kinetics. Alteration of the balance between proliferation and apoptosis is associated with cancer. The cancer cell lines were incubated with lycopene (3 μM) for 96 h. An increase in apoptosis was observed in four cell lines (T-84, HT-29, MCF-7 and DU145) treated with lycopene (Figure 3A). Most cancer cell lines that showed an increased number of cells in G2/M phase after treatment with lycopene for 96 hours (HT-29 and DU145), were also the ones with increased apoptosis (3.5 and 4 fold, respectively). This was not the case of HepG2 cells (Table 2) and cell specific differences in this regard should be studied further. In addition, lycopene increased 2-fold apoptosis in MCF-7 and T84 cells (p > 0.05). The other cell lines showed no significant difference when compared to untreated cells (p > 0.05) (Figure 3A).

To confirm the apoptosis-inducing effect of lycopene on cancer cell lines, we stained cells with DAPI. In contrast to control cells, which had very little condensed or fragmented chromatin, the majority of lycopene-treated cells displayed apoptotic features, including condensed nuclei, membrane blebbing, and nuclear fragmentation (Figure 3B and 3C).

## Discussion

The present study provided several sets of information on the inhibitory effect of lycopene on viability and on cell cycle arrest in eight different cancer cell lines.

Lycopene is highly hydrophobic, turning its administration in aqueous cell culture media difficult. Vehicles such as THF, DMSO, or lipid micelles have been proposed, and most studies use THF as the solvent [20-22], which can be toxic to some cell lines (including prostate cell lines), limiting the concentrations of lycopene. We used 10% water-soluble lycopene throughout this study, which gave satisfactory and reproducible results.

Several analyses on lycopene effect on cell proliferation used doses higher than the physiological range, which may not be observed in human blood or tissues. [2,17,22-24]. Our samples were treated with lycopene within the lower range of other investigations, which had already been detected in human serum [25,26].

Most studies reported the concentration of lycopene in the cell medium and did not monitor the cellular uptake of lycopene, which could be highly variable among cell lines. None of the cells types studied here is specialized in storage of lipophilic compounds including carotenoids, such as hepatic stellate cells previously studied by our group [19]. In this model, uptake of lycopene showed two phases. The first one ranged from 12 to more than 48 h, in which the initial low uptake level was maintained and was equilibrated with the output. Once the fat-storing phenotype was fully induced, the quantity of the stored lycopene increases abruptly, and a high quantity of lycopene was retained within these cells. We understand that this represents synthesis of new transporters or enzymes required for processing and storage of lycopene in this cell type. In the present study, lycopene was internalized early, but remained at low levels in all cells, similar to the first phase of internalization into the fat storing liver cells.

Colon cells (HT-29 and T84) reached more rapidly higher intracellular concentrations observed in other cells at 48 h. It is to be noted that these two cell lines responded earlier to lycopene, reaching maximal decrease of viability already at 48 h. The other four cell lines that responded to lycopene reached the same level of cell number decrease only after 96 h. Colon cells normally have access to alimentary lycopene, and may be provided by molecular pathways that accelerate lycopene internalization. However, the fact that other cell types that are sensitive to lycopene reached the same inhibition levels after 48 h indicated that the lycopene uptake rate is apparently not limiting for its anticancer activity, provided the equilibrium with lycopene present in the external environment. This suggests that maintaining the equilibrium of lycopene input and output may be relevant for its effect on cancer, recommending a permanent minimal supply of alimentary lycopene.

In the present study, the cell lines that have active steroid receptors (estrogen or androgen) and are sensitive to this signaling pathway were also sensitive to lycopene-mediated inhibition of cell proliferation. Interference in this pathway that controls cell proliferation in association with retinoid receptor was associated with apoptosis and inhibition of the cell cycle progression [27,28]. Furthermore, Liu et al. [29] found that lycopene can be selectively accumulated by androgen-sensitive prostate cells and localized to the nuclear membrane and nuclear matrix, suggesting a possible role for a lycopene on receptor transport.

Many studies in mammary [14,21] and prostate cancer cells [30-32] reported that lycopene is able to act as an antitumor agent by arresting cell proliferation and/or by inducing apoptosis, the action in other organs of its beneficial action is still under debate.

Prostate cancer is the second most common cause of cancer death in American men [33]. Epidemiological evidence indicates that the consumption of tomatoes or tomato-derived alimentary products and the risk of prostate cancer are inversely correlated [34]. Tang et al. [2] found no significant inhibitory or stimulating effect on growth with DU145 cells treated with lycopene at concentrations up to 50 μmol/L for the first 24 h. However, significant inhibition was observed here in DU145 cells treated with lycopene from 48 to 96 h. Lycopene at >20 μmol/L inhibited DU145 cells at 96 h as compared with controls. The 50% inhibitory concentration of lycopene for these cell lines at 96 h was 26.6 μmol/L. These concentrations were not physiological and the present results showed a cell viability reduction of 10% only after 96 h with 1, 3 and 5 μmol.

In breast cancer MCF-7 cells lycopene inhibited cell proliferation as monitored by MTT. Lycopene at micromolar concentrations inhibited MCF-7 cell growth in a dose-dependent manner and the minimal inhibiting concentration was 3.5 μM by MTT and 5 μM by BrdU assays at 48 hours. [23,25] In our study we used similar concentration and MCF-7 decreased proliferation with 1 μM of lycopene at 48 h.

Burgues et al. [15] observed no changes in cellular proliferation by cell counting at any concentration between 0.0001–10 μM of lycopene for DU145 and A549 cells, and the only cell line that responded to lycopene by cell number reduction was the HepG2 at 1 and 10 μM doses after 24 h of incubation. In our study, HepG2 and A549 lines also decreased its proliferation at 1, 3 and 5 μM, but only after 96 h of incubation. No changes in cellular proliferation were found by Hep2 and Hela cells.

Previous studies [13,18,35] report that lycopene induced a G1/S cell cycle arrest, which is corroborated by the downregulation of cyclins, including cyclin E [36] and cyclin D1 [35,37] and/or by the upregulation of cyclin A and p27 [36].

Hwang and Bowen [18] demonstrated an interference of lycopene with the cell cycle of LNCaP cells in the G2/M-phase, although Hantz et al. [22] found that purified lycopene, at concentrations lower than those used in previous studies, did not affect the proliferation but modified the apoptosis in LNCaP cells. In breast and colon cancer models, previous studies showed that lycopene promoted cell cycle modification and increased cells in the G0/G1 phase after 24 hours of exposure [12,35,38].

In our study, lycopene was able to promote cell cycle arrest, resulting in an increase of cells in G0/G1 phase or G2/M, depending upon the tumor cell type. Whilst arrest in G0/G1 can be reverted and cells will proceed the proliferation after interruption of the treatment, the G2/M arrest leads potentially to apoptosis [39]. Lycopene-induced association of the two phenomena in HT-29 and DU45 cells may be promising for prostate and adenocarcinoma prevention or treatment.

Increased resistance to apoptosis is a hallmark of many tumor cells. The functional inhibition of specific anti-apoptotic factors may provide a rational basis for the development of novel therapeutic strategies. Therefore, apoptosis deficiency is considered to be a major cause of the therapeutic resistance of tumors in the clinic, since many chemo- and radiotherapeutic agents act through the induction of apoptosis. Our results showed a significant increase in apoptosis that followed arrest cell cycle in four cancer cell lines (HT-29, DU145, T84 and MCF-7). This may indicate that lycopene could be proposed as adjuvant in cancer chemotherapy.

Other reports demonstrated that lycopene can inhibit the proliferation of cancer cells through the induction of apoptosis in different cell lines [12,30], but only a few studies demonstrated this effect after long period of treatment of lycopene and with low doses (<5 μM). Several studies suggest an effect of lycopene in decrease cyclin D1 and phospho-AKT levels and by an increase in p21, p27 and p53 levels and in Bax: Bcl-2 ratio [12].

We demonstrated the capacity of lycopene to inhibit cell proliferation, arrest cell cycle in different phases and increase apoptosis, mainly in breast, colon and prostate cell lines after 96 h. These observations suggest that lycopene may alter cell cycle-regulatory proteins depending on the type of cancer and the dose of lycopene administration. Taken together, these data indicated that the potential anticancer effect of lycopene was cellular type, time and dose-dependent.

## Materials and methods

### Reagents

*All-trans* lycopene was purchased from Sigma Chemical Company (St. Louis, MO, USA). Water-soluble (WS) lycopene (10%) was provided by Roche (Rio de Janeiro, RJ, Brazil). Dulbecco’s cell culture medium and bovine serum albumin were obtained from Sigma, and fetal bovine serum (FBS) from Laborclin (Campinas, SP, Brazil). Tissue culture flasks and cell scrapers were obtained from Nunc (Roskilde, Denmark). All the chemicals were of analytical grade.

### Cell culture and treatment protocol

All the cells lines were obtained from the Rio de Janeiro Cell Bank that has certified their identity and quality (Inmetro, Rio de Janeiro, RJ, Brazil). Human prostate cancer cells (DU145), human colon adenocarcinoma cells (HT-29), human cervical cancer cell line (Hela), breast cancer cell line (MCF-7), human liver carcinoma cells (Hep-G2) and human laryngeal carcinoma (Hep-2) cells were plated 25 cm^2^ tissue culture flasks, 5.0 × 10^6^ cells / flask, and maintained routinely in the Dulbecco’s medium supplemented (DMEM) with 10% fetal bovine serum (FBS) and 2 g/L HEPES buffer, pH 7.4, under 5% CO_2_ atmosphere. Human colon carcinoma cells (T-84) and human lung adenocarcinoma-derived cells (A549) were maintained in DMEM:HAM-F12 containing 10% FBS and 100 U/ml penicillin. Cells were passaged at 70–80% confluence, about twice a week by trypsinization. For each experiment, all the cells were seeded at 10^4^ cell/cm^2^ in 6 and 96 multiwell plates for cell cycle and cell proliferation analyses, respectively. After 24 hours, the culture medium was changed and each concentration of lycopene (WS) dissolved in water at 50 °C within a range from 1 to 5 μM. The controls were included on each plate. The cells were then incubated for 48 and 96 hours with daily medium replacement [40].

### Cell viability assay

The status of cancer cell lines viability was determined by MTT assay (Amresco, USA). Exponentially growing HSC were adjusted to 1.0 × 10^4^ cells/cm^2^ with DMEM, plated in 96-well plates (Corning, USA) at 200 μL/well and then incubated for 12 h according to routine procedure. After being treated with lycopene (1–5 μM) and incubated for 48 h and 96 h (5 wells for each sample), 20 μL/well MTT (5 g/L) was added to each well. The medium was then removed after 4 h incubation and 100 μL/well sodium dodecyl sulfate (SDS) was added to dissolve the reduced formazan product. Finally, the plate was read in an enzyme-linked immunosorbent microplate reader (Bio-Rad 2550, USA) at 490 nm. The cellular proliferation inhibition rate (CPIR) was calculated using the following formula: CPIR = (1–average A value of experimental group/average A value of control group) × 100%.

### Cell cycle analysis

Cells were rinsed briefly with calcium- and magnesium-free phosphate-buffered saline (PBS) and detached with trypsin at room temperature. After centrifugation, as the cells were washed twice with PBS, 1 × 10^6^ cells were resuspended in 1.0 mL ice-cold VindeLov solution [41], containing 0.1% Triton X-100, 0.1% citrate buffer and 0.1 mg/ml RNase and 50 μg/mL propidium iodide (Sigma Chemical Co., St.Louis, MO). After 15 minutes incubation, the cell suspension was analyzed for DNA content by flow cytometry using a FACSCalibur flow cytometer (Becton Dickinson, Mountain View, CA). The relative proportions of cells with DNA content diploid G0–G1 (2n), S phase (>2n but <4n), and G2/M phase (4n) were acquired and analyzed using CellQuest and WinMDI 2.9, respectively. The percentage of cell population at a particular phase was estimated with EXPO32 V1.2 Analysis software. Cell dissociation procedure does not affect fluorescence under the experimental conditions that were used in this study or in any others of which we are aware.

### Apoptosis assay (morphological analyses of DAPI-stained cells)

Human cancer cells lines (1.0 × 10^4^ cells/cm^2^) were treated with lycopene at a concentration of 3 μM in 96-well plates. After 96 h of incubation, cell death was detected by TdT-mediated dUTP terminal nick-end labeling kit (TUNEL, ApopTag® Plus Fluorescein in Situ Detection Kit, Chemicon Int. Inc, Temecula, CA). Nuclei were counterstained with 1 μg/mL DAPI during 3 min. Slides were examined in a fluorescence microscope (Axiophot, Zeiss) and images were acquired with AxioCam Hrc digital camera. Apoptotic data are reported as percentage apoptosis, obtained by determining the numbers of apoptotic cells *versus* the total number of cells. For each sample, a minimum of 3 counts involving a minimum of 100–200 cells/count were scored. Apoptotic data are presented as the mean ± SD for three independently performed experiments.

### Statistical analysis

The presented data are mean values ± standard error of three independent experiments done in duplicate (n = 6). Statistical comparisons were carried out by ANOVA and post hoc Tukey’s test using Graph Pad Prism 4.0 and Statistical 6.0 program. The differences were considered significant when *p* < 0.05.

## Competing interests

The authors declare that they have no competing interests.

## Authors’ contributions

TAJ, OFL, MNB, MGA and BR performed experiments and summarized the data; TTA, MRB and BR designed experiments; TAJ, MRB and BR wrote the paper; all authors have read and approved the final manuscript.
